# Acute Onset of Movement Disorders Accompanying Common Emergency Room Neurologic Disease Processes

**DOI:** 10.7759/cureus.35924

**Published:** 2023-03-09

**Authors:** Jennifer Chapman, Michael Horowitz, Ronnie Bond, Janae Fry, Christy Joesph, Yousef Al-Saghir

**Affiliations:** 1 Emergency Medicine, HCA Florida Orange Park Hospital, Orange Park, USA; 2 Neurosurgery, HCA Florida Orange Park Hospital, Orange Park, USA; 3 Neurology, HCA Florida Orange Park Hospital, Orange Park, USA; 4 Cardiology, HCA Florida Orange Park Hospital, Orange Park, USA

**Keywords:** aphasia, neoplasm, thrombectomy, athetosis, chorea, hemiballismus, dyskinesia, stroke

## Abstract

Emergency departments (EDs) of hospitals accredited for trauma and/or comprehensive stroke care treat a large volume of high-acuity patients. In this fast-paced environment, the primary focus is appropriate triage, rapid stabilization, diagnosis, and acute intervention for life-threatening conditions such as cerebral vascular accident (CVA). However, this approach may result in subtle or atypical neurologic signs and symptoms being overlooked. Often, these oversights are innocuous in terms of their influence on overall patient outcomes. They are, in the vernacular, “of academic interest only”*.* These cases provide ED clinicians with a unique opportunity to witness signs and symptoms not classically associated with common neurologic maladies. These unusual manifestations may be fleeting as they often either resolve with intervention or are overshadowed by progressive clinical decline. If such findings are recognized, they can at a minimum provide fascinating insights into neuroanatomic function. At a maximum, early recognition can influence immediate treatments and long-term outcomes.

We report three ED patient presentations that shed light on functional neuroanatomical pathways and, in one case, significantly affected a patient’s immediate algorithmic care. Two such cases involved acute middle cerebral artery distribution ischemic strokes, which typically present with focal contralateral sensorimotor and potential language deficits. Such events less commonly initially present with involuntary motor movements (dyskinesias). Failure to recognize these less common ictal signs may delay appropriate ED intervention or yield etiologic misdiagnoses. A third case involved a loss of consciousness ictal event secondary to a frontotemporal lobe tumor. This case presented with aphasic stroke-like symptoms along with new acute orofacial dyskinesias.

Imaging before and after medical, surgical, and endovascular intervention provided important clinico-anatomic lessons. Furthermore, proposed neurophysiologic mechanisms and review of pertinent literature are provided.

## Introduction

Early detection of acute ischemic stroke is essential to timely medical and neuro-interventional treatment. Delay in diagnosis or missed diagnosis may cause patients to fall outside of the treatment window for interventions such as thrombolytic therapy and thrombectomy. For this reason, most emergency departments (EDs) have specific, timed stroke protocols. Patients who present with atypical stroke symptoms, however, may not trigger initiation of stroke protocols, thereby leading to delay in diagnosis. It is important for ED physicians to include ischemic stroke in the initial differential diagnosis for patients who present with involuntary motor movements. This report describes three such case presentations, as well as their supportive imaging and outcomes following medical and endovascular interventions. Proposed neurophysiologic mechanisms and review of pertinent literature are also provided.

## Case presentation

Case 1

A 70-year-old right-handed female with a modified Rankin score (mRS) 1 presented to the ED on a Sunday evening with a witnessed, unprovoked fall. Her Glasgow Coma scale (GCS) was 15 without gaze preference and expressive or receptive aphasia. Speech was dysarthric. The patient was moving all four extremities yet displayed flamboyant new onset hemichorea of her face, mouth, and left arm along with occasional left upper extremity ballismus. National Institutes of Health Stroke Scale (NIHSS) score and Vision, Aphasia, Neglect (VAN) score were difficult to assess. Family members stated that several years earlier, the patient had a period of oral dystonia of unknown etiology, which had gradually resolved. They denied any history of upper or lower extremity movement disorders.

**Figure 1:**
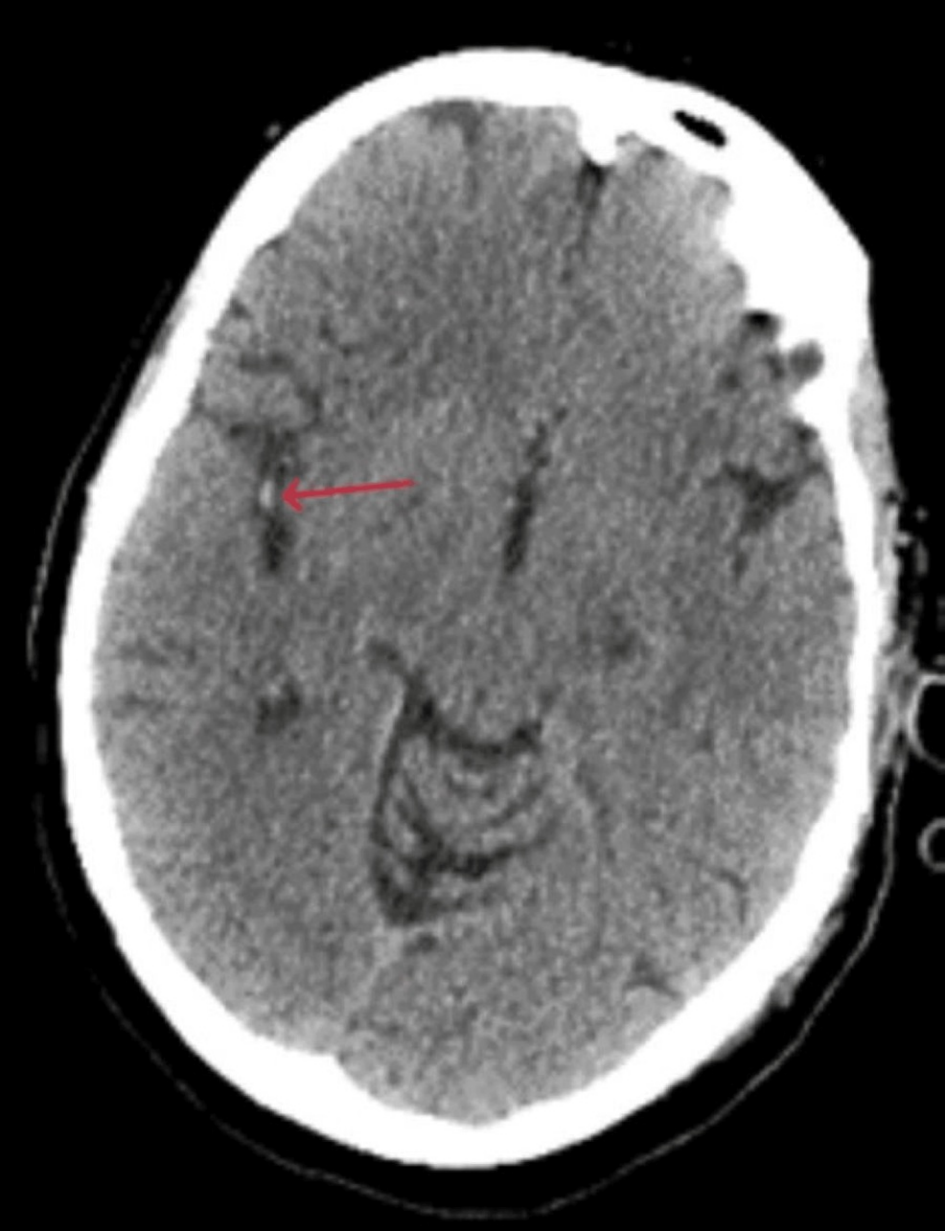
Axial brain CT showing right M2 division hyperdensity (red arrow) suggestive of arterial occlusion

**Figure 2:**
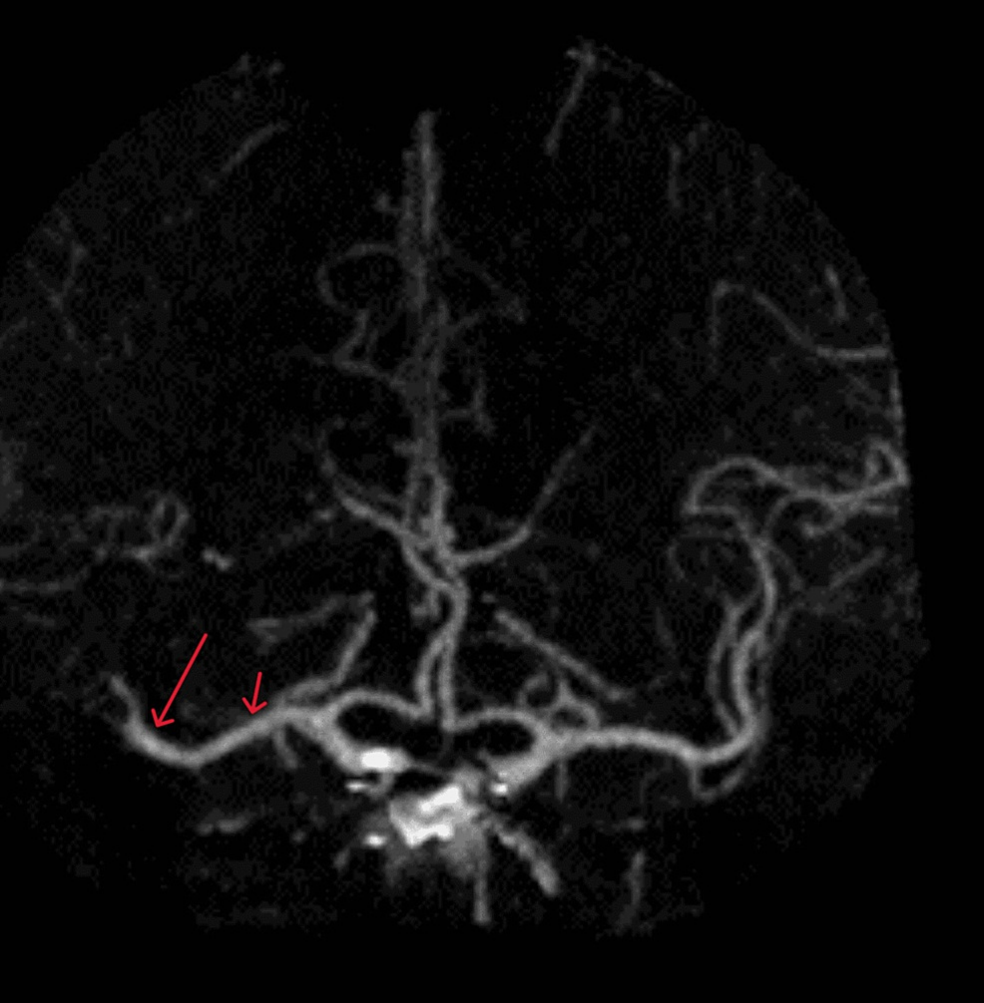
CT arteriography showing M2 occlusion (long red arrow) and lenticulostriate patency (short red arrow)

**Figure 3:**
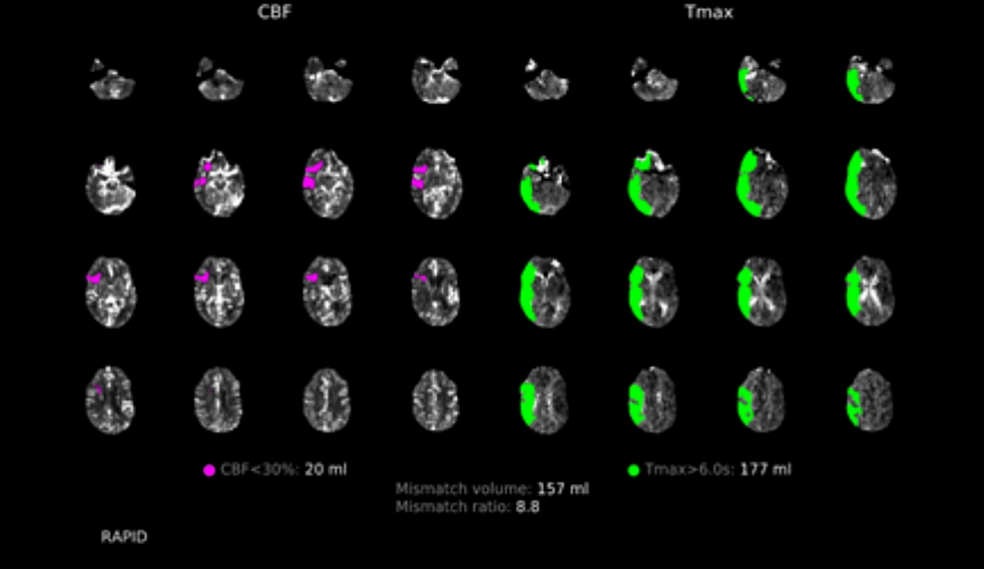
CT perfusion scan showing large penumbra:core mismatch

Brain CT without contrast revealed an Alberta Stroke Program Early CT Score (ASPECTS) of 10 with a subtle hyperdensity involving a right middle cerebral artery (MCA) M2 branch (Figure 1). CT arteriography (CTA) confirmed a superior right M2 division occlusion just distal to the right MCA M1 bifurcation along with opacification of right lateral lenticulostriate vessels (Figure 2). Brain CT perfusion showed right MCA distribution abnormalities with 20-mL cerebral blood flow < 30%, 177-mL Tmax > 6 seconds, penumbra:core 8.85, and mismatch of 157 mL (Figure 3).

**Figure 4:**
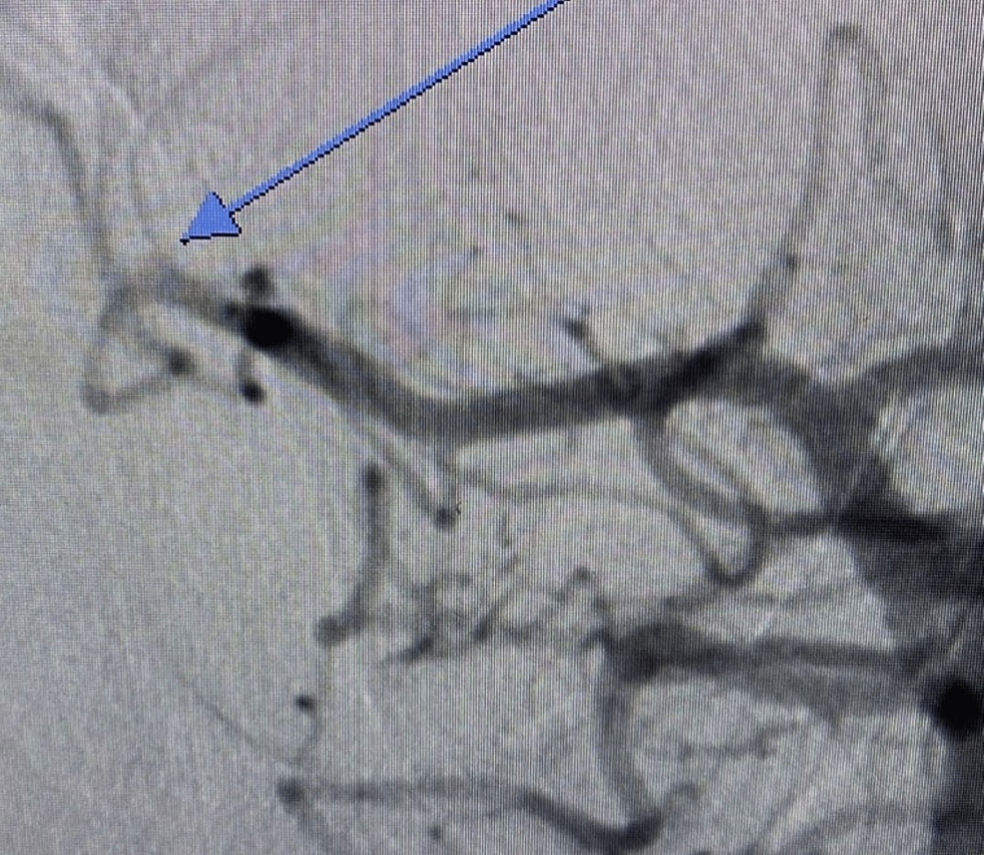
Anteroposterior internal carotid artery cerebral arteriogram showing occluded right superior M2 branch of the right middle cerebral artery (long blue arrow)

**Figure 5:**
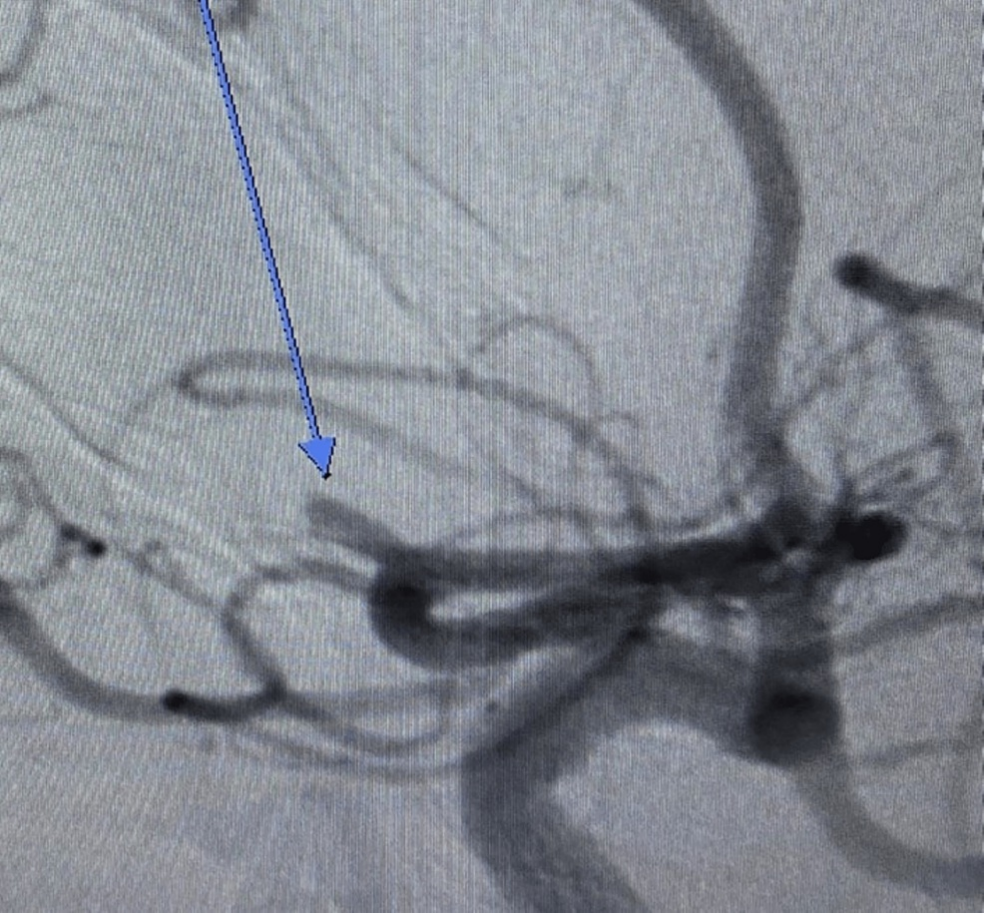
Oblique internal carotid artery cerebral arteriogram showing occluded right superior M2 branch of the right middle cerebral artery (long blue arrow)

**Figure 6:**
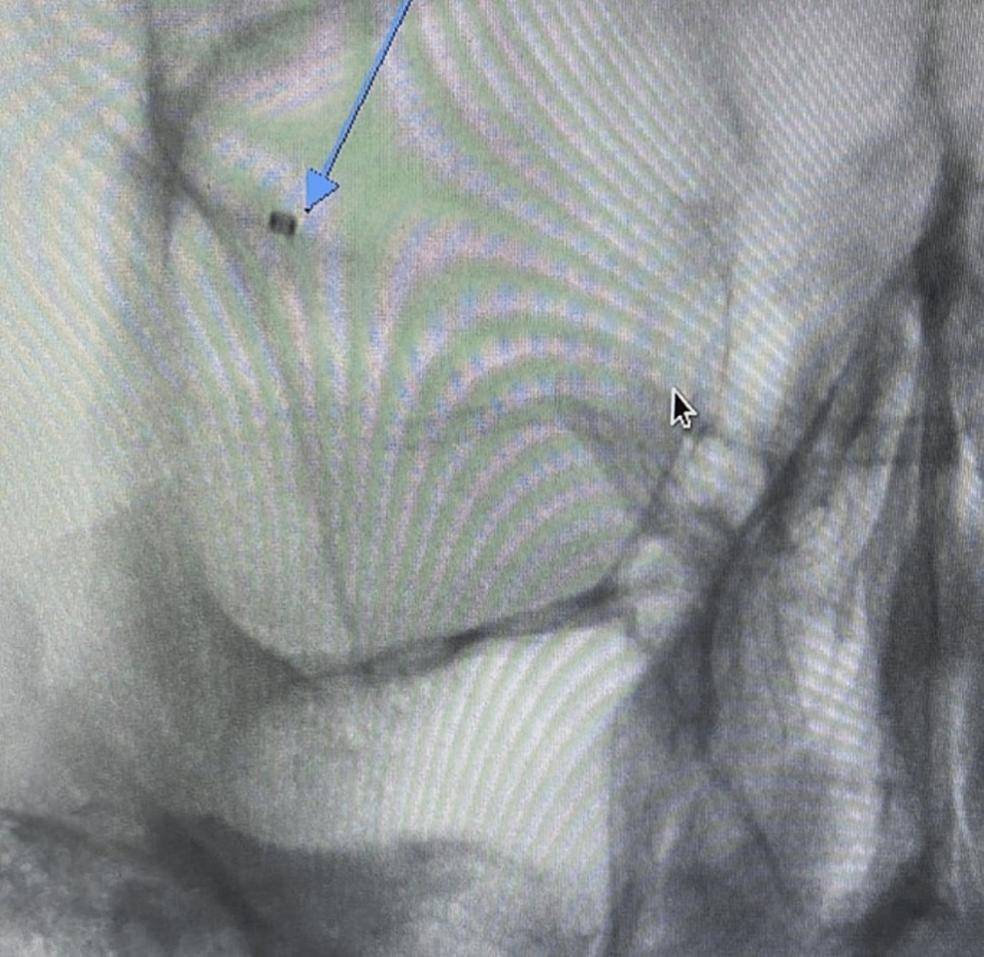
Anteroposterior CT arteriogram showing the aspiration catheter in the occluded M2

**Figure 7:**
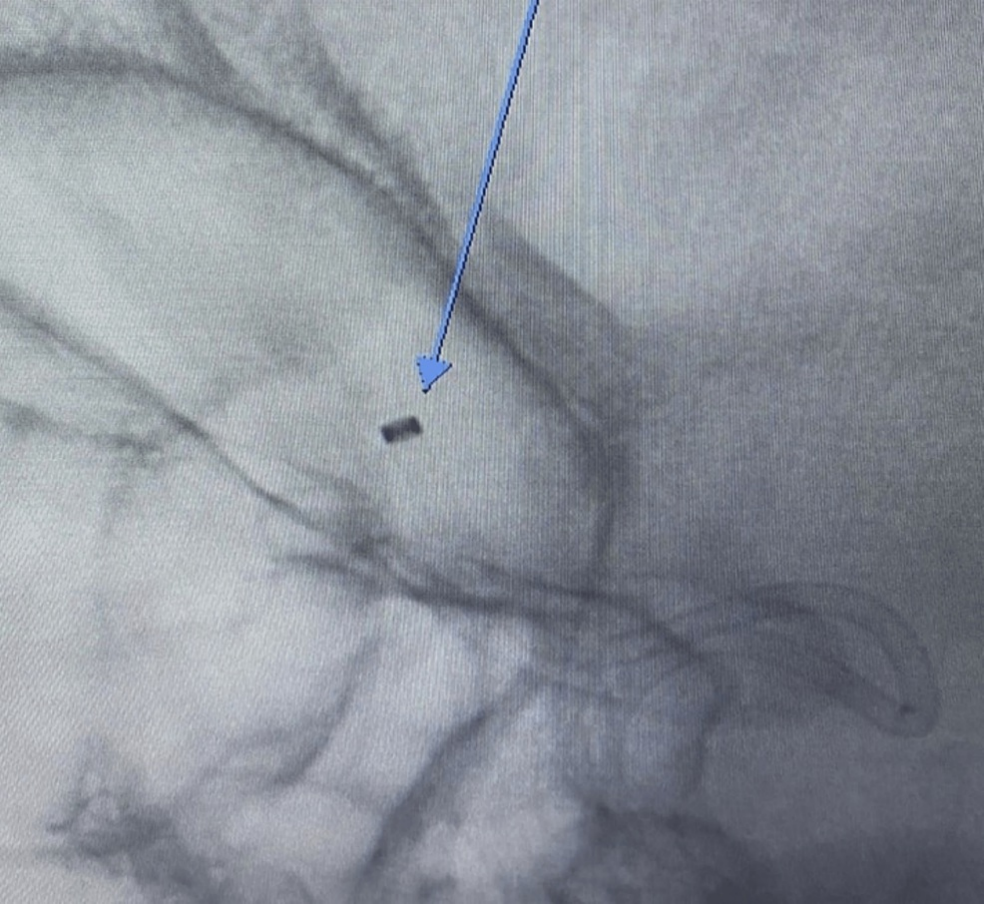
Oblique CT arteriogram showing the aspiration catheter in occluded M2

The patient received 9 mg of intravenous (IV) alteplase (TPA) followed by a 60-minute 81-mg infusion. Due to the mismatch ratio (>1.8) and mismatch volume (>15 mL), the patient was transferred to the biplane angiography suite where a right superior M2 MCA occlusion (Thrombolysis in Cerebral Infarction [TICI] 0) was identified (Figures 4-7) and opacified right lateral lenticulostriate arteries were noted. Mechanical thrombectomy was performed.

**Figure 8:**
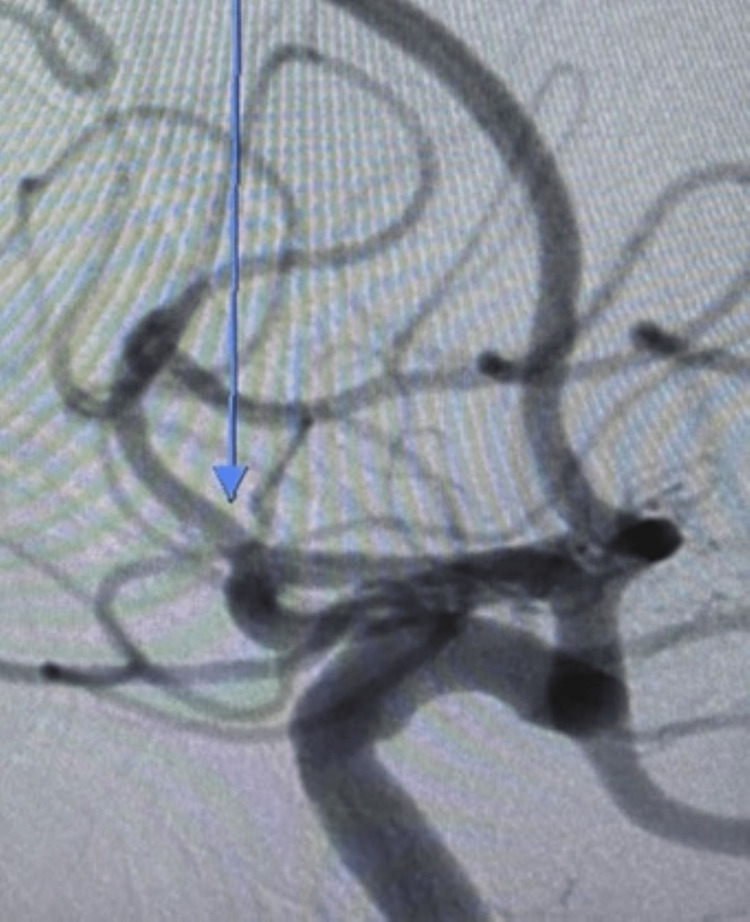
Top left image showing patent right M2 following thrombectomy

**Figure 9:**
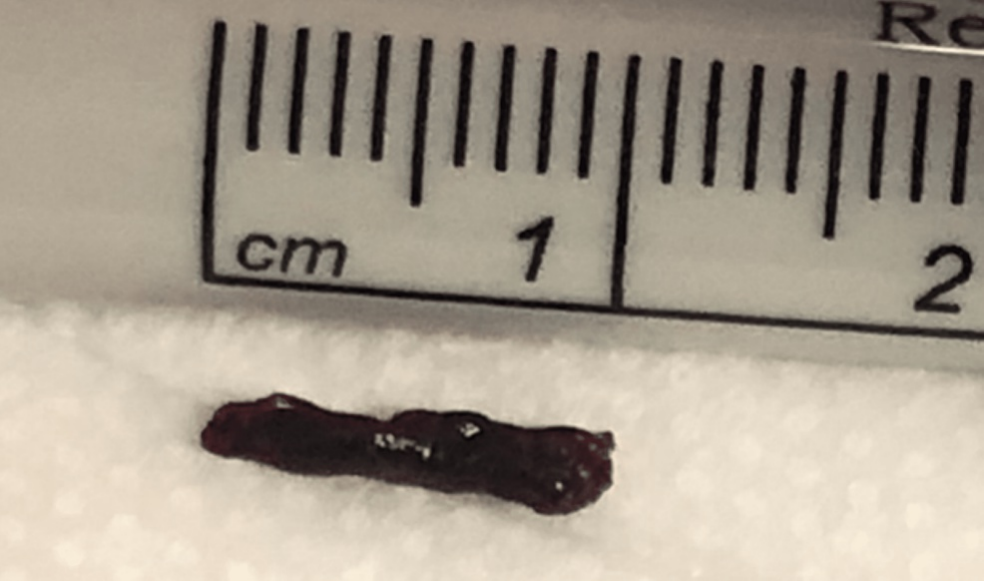
A 10-mm thrombus extracted from M2

Post-thrombectomy revascularization after two separate aspirations was TICI 3 in the M2 distribution (Figure 8). A 10-mm organized thrombus was extracted (Figure 9).

Treatment timeline was as follows:

· Door to head CT completion: 23 minutes

· Door to brain computed tomography perfusion (CTP) completion: 92 minutes

· Door to brain CTA completion: 114 minutes

· Door to needle/infusion IV TPA: 139 minutes

· Door to right common femoral artery sheath access: 237 minutes

· Door to TICI 3 revascularization: 272 minutes

All time points exceeded our accepted service normative goals because of the unusual presenting clinical examination that was not initially attributed to an ischemic event. However, once athetoid movements were identified, the patient was evaluated by the on-call stroke neurologist who, after learning of the right MCA density on a non-contrasted head CT, suggested the CTP and CTA.

Head CT performed 18 hours after IV TPA administration/thrombectomy showed acute infarct of the right frontal operculum, anterior insula, and anterior external capsule. Central to the area of infarction, a small focus of mild cortical hyperdensity likely reflecting petechial micro hemorrhage was seen. Head MRI performed 48 hours after IV TPA showed acute right MCA distribution infarcts. Foci of susceptibility within the anterior insula, frontal operculum, and anterior right external capsule indicated hemorrhagic transformation. Acute infarcts of the right frontal operculum, anterior insula, and anterior aspect of the external capsule were seen, as were cortical petechial microhemorrhage.

Within 48 hours of IV TPA/suction thrombectomy, the patient’s choreiform movements nearly completely resolved, and the NIHSS score was 2 with slight left central facial weakness. Cardiac evaluation using transthoracic echocardiography (ECG), telemetry, and cardiac markers was normal except for newly diagnosed paroxysmal atrial fibrillation.

Case 2

An 80-year-old male was admitted to the hospital with acute onset right-sided weakness that started one day prior to admission. His past medical history was significant for chronic atrial fibrillation on apixaban; however, due to a recent elective cardiac ablation, apixaban was being held due to his neurologic event. The patient's family revealed that upon awakening from his ablation, he demonstrated flailing, uncontrollable movements in his right arm, and slurred speech. They were told in ED that these anomalies were related to anesthesia, and thus the patient was sent home. When the uncontrolled ballistic movements progressed to involve both the right upper and lower extremity, the patient returned to the ED for stroke evaluation. Examination revealed flailing right extremity movements with jerking of the arm and inability to extinguish movements. On initial examination in the ED, he displayed ballistic movements of his right upper and lower extremities. Vital signs and blood glucose level were within normal limits. The duration and characterization of abnormal movements were inconsistent with epileptiform etiology. Right upper extremity strength was graded 2/5 and right lower extremity strength was graded 3/5. Gait was not accessed due to fall risk. His NIHSS score was 6. He was not considered a TPA candidate as his last known normal was > 4 hours and he had recently undergone cardiac ablation. Subsequent neurologist examination documented 3/5 right upper and lower extremity hemiparesis and non-rhythmic jerking/flailing movements of the right upper extremity described as low-volume hemi-ballistic movements. There was no dysmetria or dysdiadochokinesia.

**Figure 10:**
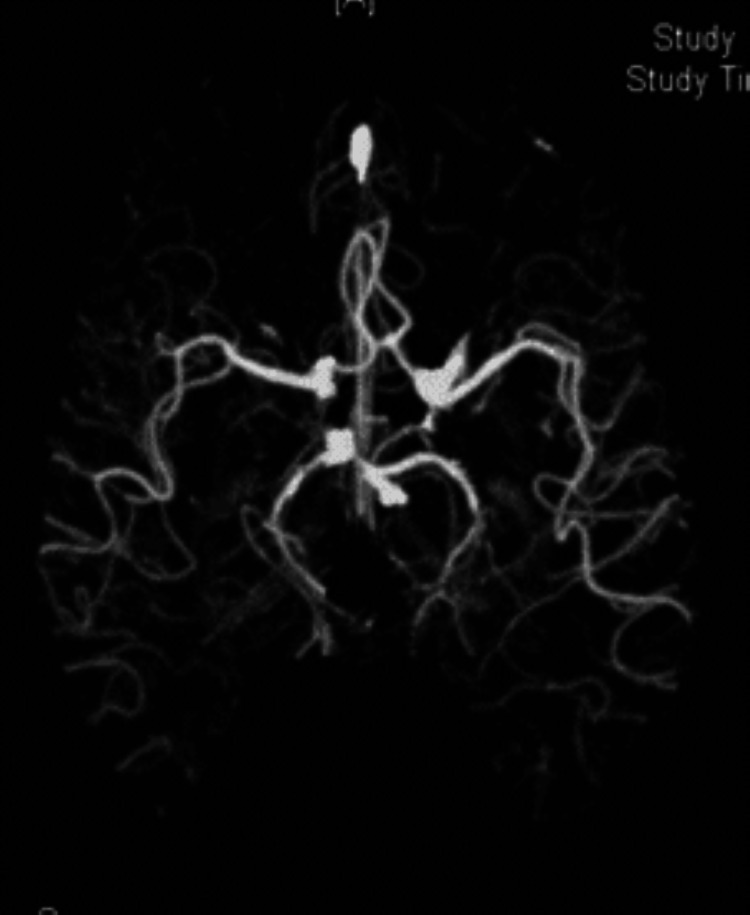
CTA showing opacification of the anterior and posterior circulation with no visible large or medium diameter vessel occlusions

Initial head CT demonstrated an acute 6 x 5 x 3 cm left parietal and posterior temporal lobe infarct. Head and neck CTA revealed moderate stenoses at the origin of each patent vertebral artery (VA), mild/moderate right and left posterior cerebral artery atherosclerotic disease, 50% left cervical internal carotid artery (ICA) and 45% right cervical ICA stenoses, and approximately 72% and 75% stenosis of the right and left ICA’s clinoidal segments, respectively. The anterior cerebral arteries and MCAs were bilaterally patent (Figure 10).

**Figure 11:**
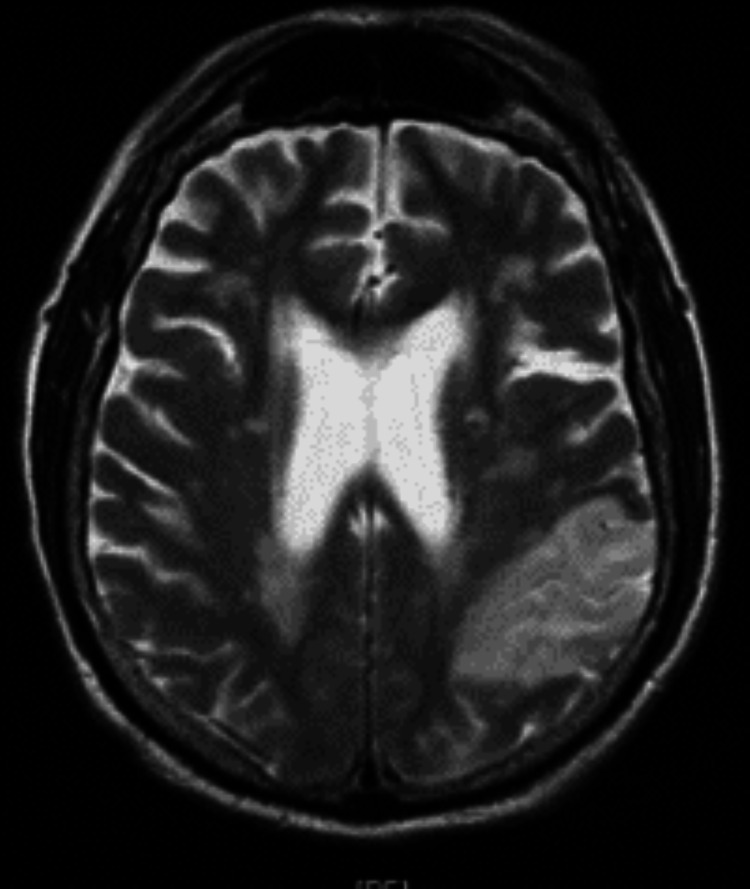
T2-weighted MRI showing T2 signal abnormality

Due to motion, CTP was non-diagnostic. Brain MRI (Figure 11) demonstrated restricted diffusion within the left posterior MCA territory involving the left parietal lobe, left posterior frontal lobe, and posterior-lateral temporal lobe, compatible with a known evolving subacute infarct. There was superimposed petechial hemorrhage but no space-occupying hematoma. There was mild associated local mass effect but no midline shift or herniation. Of note, there were no diffusion-weighted imaging (DWI) abnormalities identified in the basal ganglia (BG), thalamus, or extreme capsule.

The patient was admitted and initiated on aspirin 81 mg daily and atorvastatin 80 mg daily. He was resumed on apixaban 5 mg twice per day after observation of the petechial hemorrhage. On admission day 2 (day 3 post-ischemic ictus), hemi-ballistic movements spontaneously resolved. He continued with mild right-sided weakness, though there was marked improvement in right upper extremity strength at 4+ out of 5 and right lower extremity at 5/5. He was discharged home in stable/improved condition on post-ictus day 5.

Case 3

**Figure 12:**
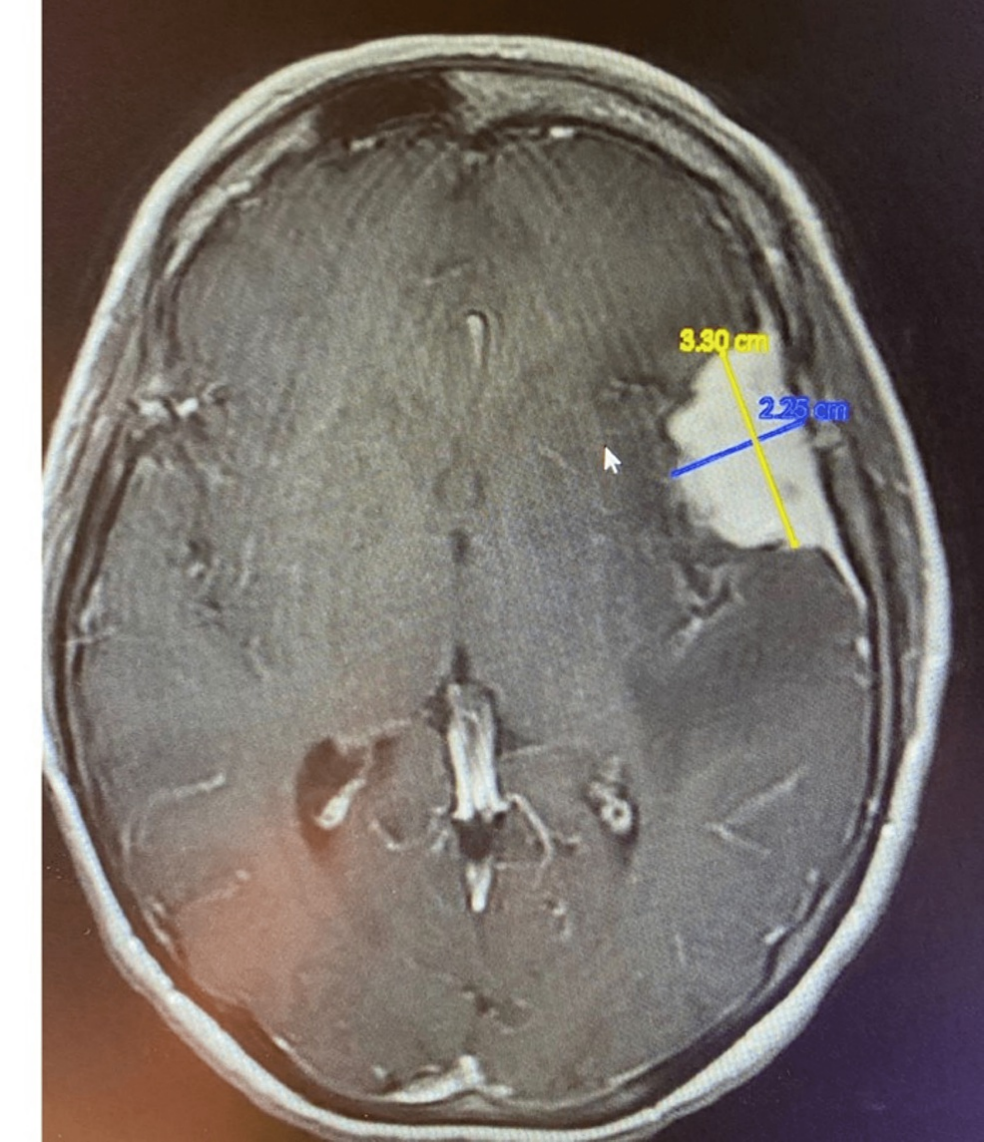
MRI showing left anterior middle fossa meningioma

**Figure 13:**
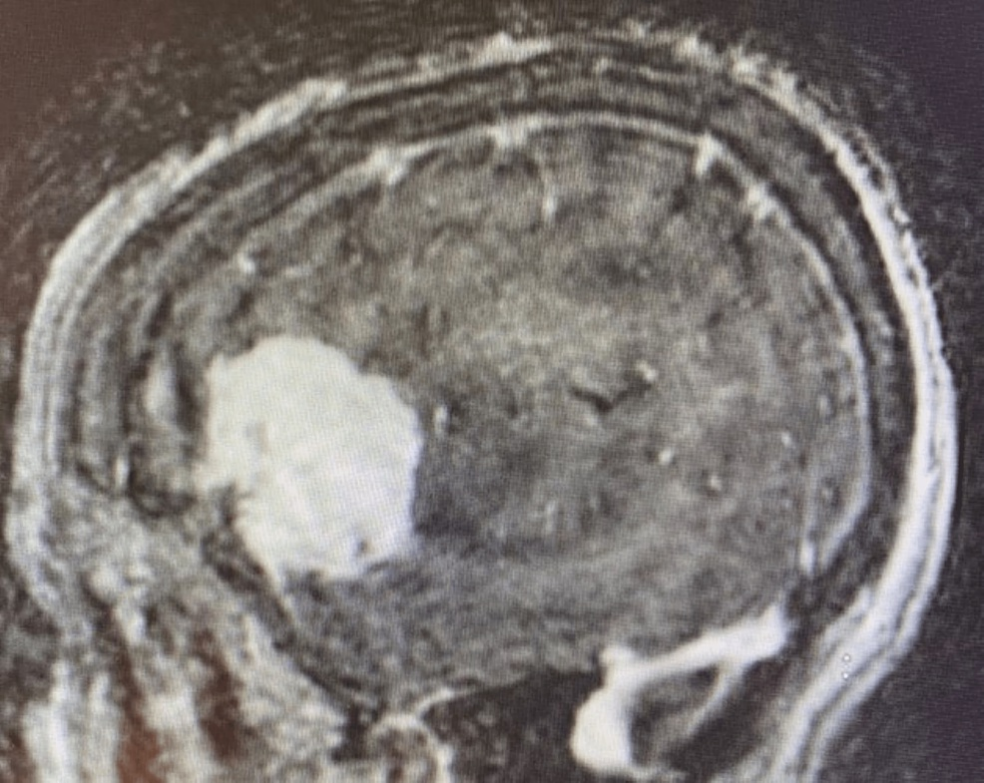
MRI showing left anterior middle fossa meningioma

**Figure 14:**
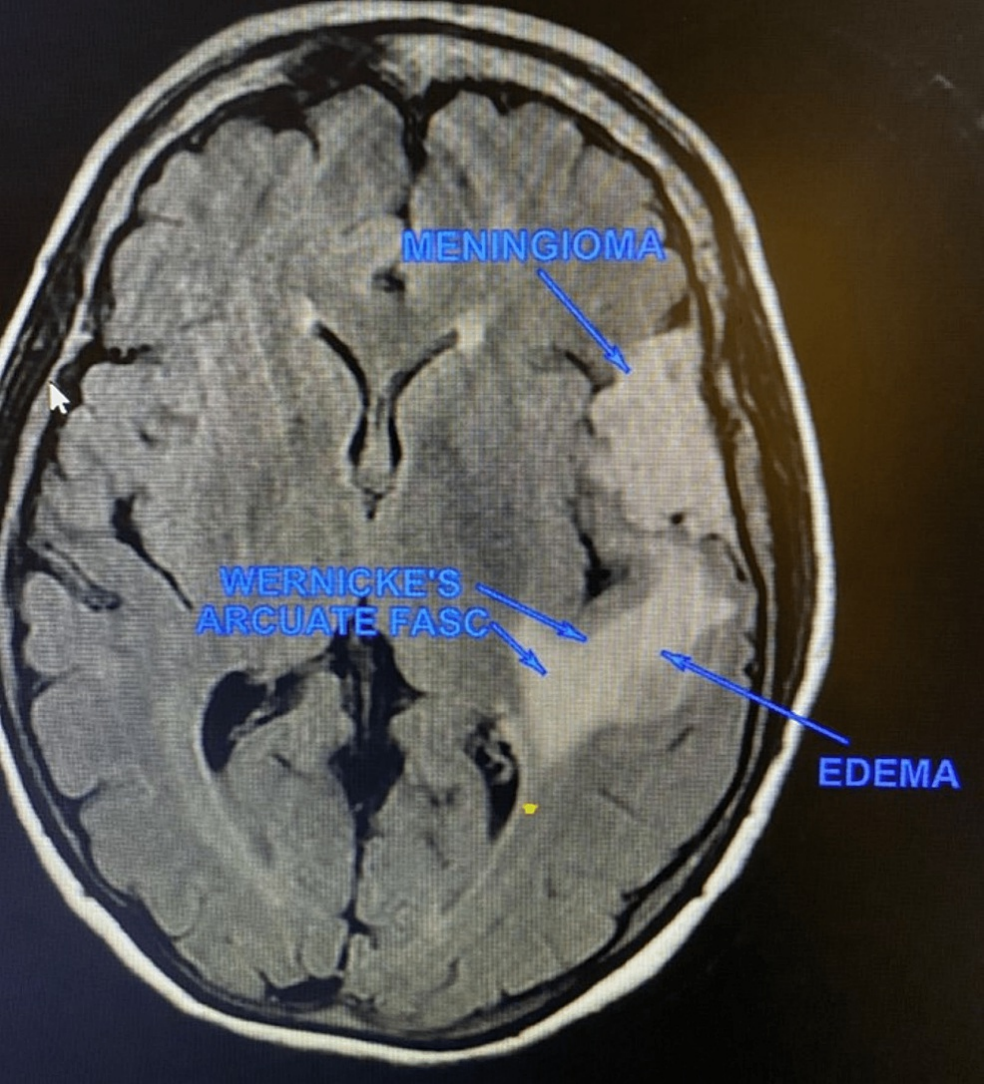
Vasogenic edema extending posteromedially to involve Wernicke’s area

A 56-year-old female without significant past medical history collapsed at home following a questionable de novo generalized tonic-clonic seizure. Upon presentation to the ED, she was awake and alert. She displayed expressive aphasia and orofacial dyskinetic movements involving the right side of her mouth, lips, and tongue. Initial diagnosis was ischemic stroke, yet a CT and CTA of the brain demonstrated a 4-cm partially cystic, intensely contrast-enhancing dural-based lesion arising at the level of the left lateral pterion such that it exerted mild invaginating mass effect on the underlying fronto-temporal cortex with no accompanying midline shift nor mesial temporal uncal herniation. Contrast MRI subsequently revealed the aforementioned dural-based mass with a dural tail along with considerable peripheral vasogenic edema that extended posteriorly and medially (Figures 12-14).

Both imaging modalities demonstrated the tumor to be significantly anterior to the left angular and supramarginal gyri, although vasogenic edema did involve the adjacent left parietotemporal gray and subcortical white matter as well as the extreme capsule (Figure 6). The patient, who remained awake and purposeful, was assumed to be having ongoing focal motor seizures and was administered 1,000 mg IV levetiracetam along with 20 mg IV dexamethasone. Orofacial movements and expressive aphasia continued. She retained the ability to follow simple midline commands, but she could not speak, repeat, or make phonetic sounds. Reading was not tested. In addition to the above, the patient had difficulty swallowing her saliva, and when asked to protrude her tongue, she did so in a dyskinetic manner with the tongue moving about randomly as it was held outside the mouth. She was administered IV dexamethasone (20 mg IV bolus followed by 4 mg IV every 6 hours) and IV levetiracetam (1,000 mg bolus followed by 500 mg twice a day).

**Figure 15:**
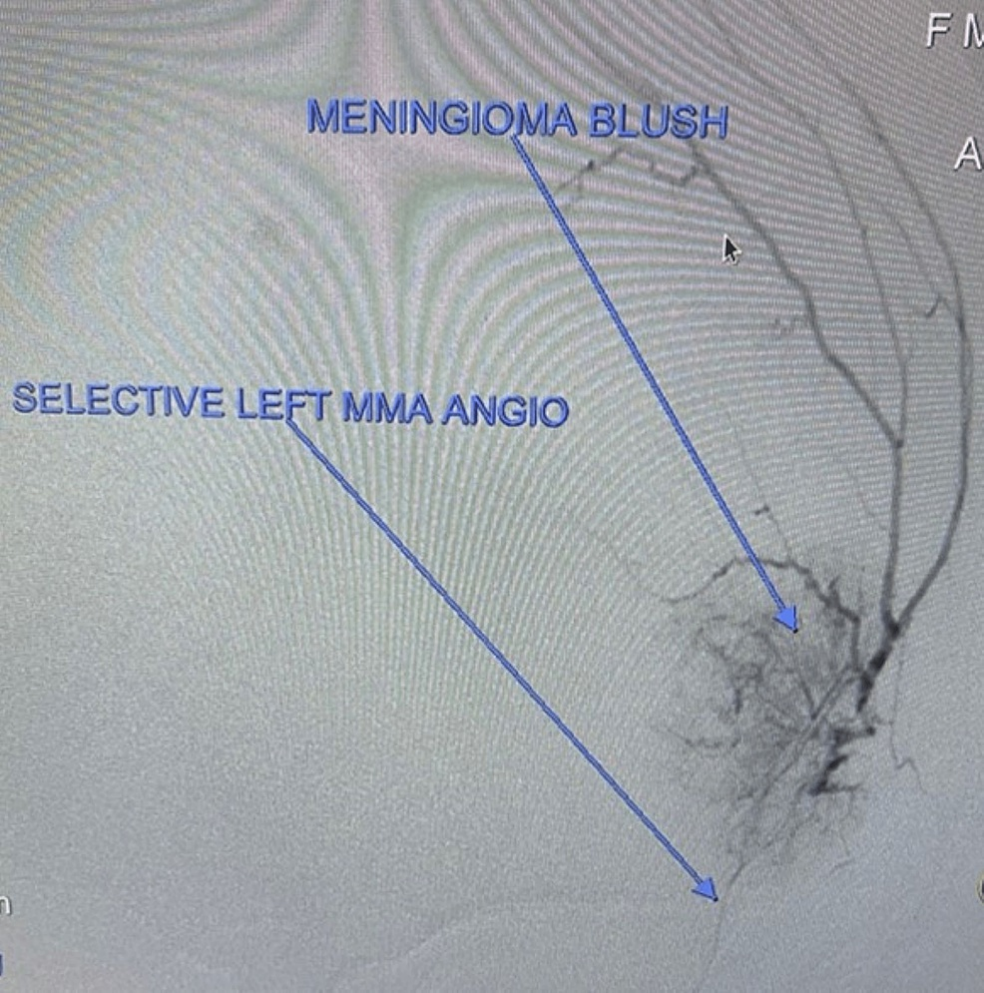
Pre-embolization arteriogram demonstrates middle meningeal artery blood supply

Over the next 36 hours, the patient had improvement in her condition with improvement of her expressive aphasia and dyskinesias. Swallowing study revealed inability to properly swallow. At the 36-hour mark, she underwent preoperative embolization of the presumed meningioma’s middle meningeal artery (MMA) blood supply (Figure 15), and at the 60-hour mark, she underwent tumor resection. Final histopathology revealed the diagnosis of mixed meningothelial and angiomatous meningioma.

The patient was discharged to rehabilitation where she gradually recovered her speech and swallowing.

## Discussion

Neuroanatomy

Involuntary Movements

The modulation of motor function is complicated and remains incompletely understood with a variety of central nervous system lesions causing similar movement disorders [1]. Basic understanding, however, designates the BG as the feedback center for human motor activities [2]. A simplified explanation holds that motor signals from the neocortex through the thalamus, striatum, and pallidum, where they are modified by inhibitory and excitatory inputs prior to returning to the cortex [1]. Thalamic or BG injury can occur due to stroke and may result in development of athetosis, chorea, ballismus, dystonia, myoclonus, asterisks, tremor and Parkinsonism [2].

According to Handley et al. [1], while hemichorea, dystonia, and hemiballismus may develop due to lesions of the striatum (caudate and lenticular nuclei [globus pallidus and putamen]), these movement disorders can also occur following thalamic, subthalamic, internal capsule, corona radiata, frontal lobe, parietal lobe, external capsule, pontine, and temporal lobe injuries. Huntington’s disease, which can manifest with involuntary, unwanted movements in small orofacial muscles leading to dysarthria and dysphagia, has been shown using MRI to have extensive involvement of the corpus callosum and external/extreme capsules. This may indicate that disturbances of these interhemispheric white matter tracts can cause patients to exhibit dyskinetic movements [1]. Such findings may be relevant to our patient (case 3) who presented with orofacial dyskinesias, tumor-induced vasogenic edema, and compression/distortion of the external and extreme capsules.

Athetosis is rarely reported as an isolated disorder but is usually accompanied by hemichorea with or without hemiballism. Other accompanying symptoms can include dysarthria and difficulty with tongue movements [1]. Athetosis, rarely seen in isolation, can be pseudo-athetoid in origin due to sensory changes and abnormal proprioception, which makes it difficult for the patient to spatially localize and position a limb/joint.

Dysphasia/Aphasia

Dysphasia/aphasia is generally divided into four categories: receptive, expressive, conductive, and transcortical. For a detailed review, the reader may refer to the study by Damasio [3]. Traditionally, focus has generally been placed on the supramarginal, angular, and inferior frontal gyri, the midsagittal falcine region, and the arcuate fasciculus when explaining aphasia neurophysiology. More recent studies, however, indicate that the extreme capsule contains fiber tracts (distinct from the arcuate fasciculus) that connect the inferior frontal gyrus (Broca’s area), the middle part of the superior temporal gyrus (Wernicke’s area), and the inferior parietal lobule (angular gyrus). Such findings indicate that the extreme capsule may have a prominent role in language function [4].

With the above in mind, it is interesting to consider the extreme degree of language impairment displayed by the patient in case 3. This individual’s meningioma is located in a region that is not adjacent to either Broca’s or Wernicke’s areas, yet the patient displayed a complete inability to speak and limited ability to follow simple commands. The MRI, however, did demonstrate considerable extreme capsule compression along with significant tumor-related vasogenic edema involving the angular and supramarginal gyri and the posterior arcuate fasciculus. These imaging findings provide an interesting example of how a mass, remote from traditional critical cortical tracts, can influence neurologic function by indirectly disturbing these tracts and their efferent and afferent connections via neoplasm-induced tissue changes (edema, swelling) as well as by direct compression of other less appreciated and less well understood, yet clearly important white matter tracts such as those traversing the extreme capsule [5].

Incidence, Prevalence, and Demographics

Involuntary movement disorders (dyskinesias) such as dystonia, chorea, ballismus, and Parkinsonism are identified after 1-4% of ischemic/hemorrhagic strokes [2]. Most of these anomalies become evident after the initial motor deficits (weakness) begin to improve [2]. Nevertheless, some clinicians have identified choreaform abnormalities within few hours of stroke onset [6]. The Lausanne Stroke Registry identified 29 of 2,500 patients (1.1% prevalence; 0.08% incidence) with acute or delayed movement disorders after their first stroke. Of this subset, 38% displayed hemichorea-hemiballism (0.4% of registry patients), with chorea affecting older patients and dystonia affecting younger patients. Women and men are equally at risk. While the mean age in this registry was 70 years, others have found mean ages closer to 60 [7]. These findings are supported by Suri et al., who reviewed 284 published cases of movement disorders after hemorrhagic and ischemic stroke and found that 17.4% of such disorders were characterized by chorea [6]. Handley et al., after reviewing publications between 1966 and 2008, found only 156 case reports describing post-stroke movement disorders. Chorea was the most common, with a mean onset of 4.3 days following the ischemic event [1].

Emergency Department Considerations

Between 2006 and 2014 in the USA, there were 3.9 million ED visits for stroke and 2.5 million for transient ischemic attacks [8]. The rate of ED visits for stroke symptoms grew at a rate of 25% over this eight-year period [8]. Fortunately, the increasing rate of ED stroke visits coincided with the advent of effective therapies such as IV TPA and endovascular catheter-based therapies. The growing availability of these time-sensitive therapies has prompted hospitals to aggressively focus on early stroke recognition. Rapid ED assessments aim to quickly identify patients who may benefit from emergent extracranial/intracranial arterial revascularization. Standardized stroke assessment scales have been developed to aid in this process, such as the widely used NIHSS. Additional scales include the modified NIHSS, Cincinnati Stroke Triage Assessment Tool (C-STAT), Gaze-Face-Arm-Speech-Time test (G-FAST), Prehospital Acute Stroke Severity (PASS), Conveniently-Grasped Field Assessment Stroke Triage (CG-FAST), Face-Arm-Speech-Time plus severe arm or leg motor deficits tests (FAST-PLUS), and Vision, Aphasia, Neglect test (VAN).

While these scales have proven useful to varying degrees in the early detection of acute ischemic stroke and strokes secondary to large vessel occlusion (LVO), their utility may decrease in patients with atypical neurologic signs and symptoms. In such cases, diagnosis of cerebral ischemia may be delayed or missed altogether. Given the difficulty in ED assessment of atypical neurologic symptoms, EMS personnel and ED clinicians should have a low threshold for initiating emergent stroke evaluation in any patient presenting with acute neurologic symptoms. Stroke evaluation should include non-contrasted head CT, CT arteriogram, and CT perfusion study with possible MRI. Since stroke imaging is a prerequisite for IV TPA and endovascular interventions, transfer to a higher level of care should be considered for patients received at EDs that do not have readily available sophisticated neuroimaging.

Outcomes

Patients who develop movement disorders following ischemic or hemorrhagic strokes may experience significant recovery over the ensuing 12 months. Ghika-Schmid et al. [7] noted marked improvement within two weeks of ictus in 15 (50%) of their 29 cases; 26 (90%) of their patients recovered by 12 months. Other investigators have noted similar degrees of partial or complete recovery by one year [9]. Other series, however, are more sanguine especially for non-choreiform disorders [1].

The terms used in the discussion have been defined in the Appendices.

## Conclusions

Ischemic and hemorrhagic strokes can present with a vast array of symptoms. This report documents the rare occurrence of acute/immediate onset hemiballismus, chorea, and athetosis as presenting signs of MCA ischemia. It also documents movement disorders associated with tissue distortion of the external and extreme capsules from vasogenic edema and tumor mass effect. While transient thalamic/striatal ischemia from lenticulostriate distribution oligemia cannot be excluded as a cause for some patient symptoms, it is interesting to note that brain MRI performed 48 hours following the ictal events revealed no DWI changes in these locations. This report is unique as it documents pre- and post-intervention imaging including CT, CTA, biplane digitally subtracted catheter angiography, CTP, and MRI. The combination of IV TPA and manual aspiration thrombectomy yielded successful outcomes with rapid improvement in signs and symptoms over the ensuing 24 -48 hours. This report also points to the delays in diagnosis and treatment that developed because of an atypical clinical presentation. It is our hope that this publication will remind others of the rare but potential presentation of LVO with acute movement disorder. Recognition will improve ED triage thus accelerating diagnosis and treatment.

## Appendices

**Table 1:** Definitions

Terms	
Dyskinesia	Abnormal, uncontrollable, involuntary movements
Hemichorea	Involuntary, irregular, and unpredictable movements that may appear as if the affected person is dancing, twisting, restless, clumsy, or fidgety
Ballism	Rapid large-scale flinging movements with limb rotation at the proximal joint
Dystonia	Prolonged muscle contraction resulting in twisting movements and unusual fixed postures
Athetosis	Slow, serpentine, movements usually involving the hands and feet

## References

[1] Handley A, Medcalf P, Hellier K, Dutta D (2009). Movement disorders after stroke. Age Ageing.

[2] Mehanna R, Jankowitz J (2013). Movement disorders in cerebrovascular disease. Lancet.

[3] Damasio AR (1992). Aphasia. N Engl J of Med.

[4] Makris N, Pandya DN (2009). The extreme capsule in humans and rethinking of the language circuitry. Brain Struct Funct.

[5] Kümmerer D, Hartwigsen G, Kellmeyer P (2013). Damage to ventral and dorsal language pathways in acute aphasia. Brain.

[6] Suri R, Rodriguez-Porcel F, Donohue K, Jesse E, Lovera L, Dwivedi AK, Espay AJ (2018). Post-stroke movement disorders: the clinical, neuroanatomic, and demographic portrait of 284 published cases. J Stroke Cerebrovasc Dis.

[7] Ghika-Schmid F, Ghika-Regli F, Bagousslavsky J (1977). Hyperkinetic movement disorders during and after acute stroke: Lausanne Stroke Registry. J Neurol Sci.

[8] Bedaiwi II, Alfaraj SZ, Pines JM (2018). National trends in stroke and TIA care in U.S. emergency departments and inpatient hospitalizations (2006-2014). Am J Emerg Med.

[9] Alarcon F, Zijlmans JC, Duenas G, Cevallos N (2004). Post-stroke movement disorders: report of 56 cases. J Neurol Neurosurg Psychiatry.

